# Chimeric DNA byproducts in strand displacement amplification using the T7 replisome

**DOI:** 10.1371/journal.pone.0273979

**Published:** 2022-09-19

**Authors:** Dillon B. Nye, Nathan A. Tanner

**Affiliations:** Nucleic Acid Replication Division, New England Biolabs Inc., Ipswich, Massachusetts, United States of America; Universität Stuttgart, GERMANY

## Abstract

Recent advances in next generation sequencing technologies enable reading DNA molecules hundreds of kilobases in length and motivate development of DNA amplification methods capable of producing long amplicons. In vivo, DNA replication is performed not by a single polymerase enzyme, but multiprotein complexes called replisomes. Here, we investigate strand-displacement amplification reactions using the T7 replisome, a macromolecular complex of a helicase, a single-stranded DNA binding protein, and a DNA polymerase. The T7 replisome may initiate processive DNA synthesis from DNA nicks, and the reaction of a 48 kilobase linear double stranded DNA substrate with the T7 replisome and nicking endonucleases is shown to produce discrete DNA amplicons. To gain a mechanistic understanding of this reaction, we utilized Oxford Nanopore long-read sequencing technology. Sequence analysis of the amplicons revealed chimeric DNA reads and uncovered a connection between template switching and polymerase exonuclease activity. Nanopore sequencing provides insight to guide the further development of isothermal amplification methods for long DNA, and our results highlight the need for high-specificity, high-turnover nicking endonucleases to initiate DNA amplification without thermal denaturation.

## Introduction

The study and creation of large DNA molecules is a growing area of biotechnology as synthetic biology increasingly pushes the size limits of DNA assemblies [1]. In addition to tools for creating larger synthetic DNA, long-read sequencing technologies (e.g. Oxford Nanopore, Pacific Biosciences) are continually increasing their length capabilities and enabling new avenues of investigation. Long-read sequencing offers fundamental advantages over traditional next generation sequencing methods, notably in assembling genome regions which have low complexity or are highly repetitive [2, 3]. To fully utilize the capabilities of long-read sequencing, new methods for amplification of large DNA molecules must be developed. Current amplification technologies are limited both in size and can be prone to various types of errors, with amplified species difficult to produce over ~20 kilobase pairs (kbp). Amplification using the polymerase chain reaction (PCR) is size-limited due to template degradation resulting from the requisite near-boiling temperatures [4, 5]. Additionally, the limited processivity of the PCR polymerases and the frequent dissociation and initiation of the elongating enzyme on dissociating and re-annealing template structures produces chimeric DNA molecules [6]. Isothermal DNA amplification methods may ameliorate some of the limitation associated with PCR by utilizing more efficient replication enzymes and avoiding thermal denaturation and cycling.

Numerous isothermal DNA amplification strategies have been developed [7]. These may rely on enzymatic activities to aid in the annealing of a primer to one strand of a double-stranded DNA (dsDNA) substrate, as in recombinase-polymerase amplification and helicase-dependent amplification [8, 9]. Alternatively use of specially designed primers can facilitate initiation and subsequent extension steps, as in loop-mediated isothermal amplification and traditional strand-displacement amplification [10, 11]. Isothermal amplification methods all utilize a DNA polymerase capable of extending a primer on a dsDNA template while displacing the non-template strand and have found utility primarily as molecular diagnostic methods where short target amplification is desirable for detection speed. The only isothermal method utilized for long DNA products, whole-genome amplification by the ϕ29 DNA polymerase, produces hyperbranched networks of DNA containing a large fraction of chimeric sequences [12]. These limitations, combined with the increasing ability to manipulate and sequence long DNA, motivate the development of isothermal DNA amplification tools for longer, sequence-specific DNA targets.

The DNA replication machinery of the T7 bacteriophage has long been studied as a model replication system and could potentially serve as such an amplification tool [13, 14]. The T7 replisome is a dynamic macromolecular assembly of only four proteins: gp2.5, a homodimeric single-stranded DNA (ssDNA) binding protein; gp4, a hexameric ATPase that functions as both a helicase and a primase; gp5, a DNA polymerase; and *Escherichia coli* thioredoxin (Trx), which binds gp5 to increase the processivity of DNA synthesis [15]. The helicase gp4 encircles one strand of a dsDNA substrate and translocates in the 5’ to 3’ direction, also synthesizing RNA primers for lagging-strand synthesis. The other displaced strand is used as a template for continuous leading-strand synthesis by gp5+Trx polymerases associated with the gp4 hexamer. Because lagging-strand synthesis is discontinuous and requires additional proteins for maturation, it is of limited utility for in vitro amplification strategies and can be excluded in practice by omitting ribonucleotides and/or removal of primase activity from gp4. As illustrated in a recent cryogenic electron microscopy structure [16], the precise arrangement of gp4 and the leading strand gp5-Trx polymerase allows the assembly to function together with gp2.5 as a highly processive strand-displacing DNA polymerase.

Though the T7 replisome naturally initiates DNA replication in coordination with T7 RNA polymerase, is has been found to initiate DNA polymerization from nicks in dsDNA [17, 18]. In the canonical isothermal method strand-displacement amplification, site-specific nicks are introduced with specific primers and an associated endonuclease [11, 19, 20]. Early implementations of this method used alpha-thiophosphate nucleotides to enable DNA nicking by a restriction endonuclease, while modern strategies use nicking endonucleases (NEases) with 5–7 basepair (bp) recognition sequences [21]. NEases enable amplification strategies that eschew primers altogether, referred to as linear strand-displacement amplification (LSDA) in reference to linear amplification kinetics achieved by multiple rounds of DNA nicking and extension. Notably, a survey of DNA polymerases identified Δ28 T7 gp5, a modified form of gp5 lacking 28 residues and 3’-5’ exonuclease activity [22], as adept at initiating synthesis from DNA nicks in the presence of *E*. *coli* single stranded DNA binding protein (SSB) [23]. The high processivity of the T7 replisome, which can routinely synthesize more than 40 kb of DNA in a single binding event, could enable amplification of long DNA molecules from specified DNA nicks [24, 25].

To gain a mechanistic understanding of strand-displacement amplification reactions using the T7 replisome, we employed Oxford Nanopore sequencing. Nanopore sequencing enables sequence determination of individual amplified dsDNA molecules and permits a high-resolution interrogation of T7 replication products [26]. The linear 48.5 kbp dsDNA λ bacteriophage chromosome was selected as a substrate for reaction with three NEases and either the WT gp5 or Δ28 gp5 T7 replisome. Strikingly, the WT and Δ28 gp5 T7 replisome produce different amplicons resulting from a template-switching mechanism linked to polymerase 3’ exonuclease activity. Template switching, or the generation of chimeric DNA molecules, is prevalent in all of the T7 reactions in analogy to reactions using the ϕ29 bacteriophage DNA polymerase [27]. Nonetheless, amplification of linear dsDNA of at least 13 kbp is observed, highlighting the possible utility as well as challenges to amplification of long DNA molecules using the T7 replisome.

## Materials and methods

### Materials

T7 reactions include Tris acetate (J. T. Baker, Phillipsburg, NJ), potassium acetate (Sigma Aldrich, Natick, MA) and magnesium acetate (Sigma Aldrich). Ethylenediaminetetraacetic acid (EDTA, VWR, Radnor, PA) and Triton X-100 (American Bioanalytical, Canton, MA) were used to quench reactions or in protein purification. Except for Sequenase v2.0 (Δ28 gp5, Thermo Fisher, Waltham, MA) and unless otherwise noted, all enzymes and reagents were provided by New England Biolabs.

### Purification of T7 gp4 and gp2.5

Genes encoding for T7 gp4A’ (M64G variant of the full length 63 kDa gene product [28], hereafter gp4) and gp2.5 were cloned into the pAII17 expression vector [29]. The expression plasmid for T7 gp2.5 was constructed using the unmodified gp2.5 sequence [30] by GenScript (Piscataway, NJ). In the case of T7 gp4, a codon-optimized gene sequence was ordered from IDT (Coralville, IA) as a gBlock and cloned into the pAII17 vector using the NEBuilder HiFI DNA Assembly Cloning kit. We note that the unmodified gp4 sequence appeared to be toxic to *E*. *coli* and codon optimization was required to produce significant levels of soluble protein [31]. Transformation into NEB T7 Express *lysY/I*^*q*^ Competent cells yielded expression strains that produce high levels of soluble gp2.5 and moderate levels of soluble gp4 protein. Purifications of gp2.5 and gp4 were performed using similar strategies that were modifications of published protocols [28, 32]. Details on the protein purifications are given in the Supporting Information.

### Reaction of λ DNA with the T7 replisome and nicking enzymes

Genomic λ phage DNA (100 ng, NEB) was incubated with components of the T7 replisome and a NEase in reaction buffer (50 mM Tris acetate pH 7.9, 50 mM potassium acetate, 2 mM DTT, 3.5 mM dTTP, 1 mM other dNTPs). Components of the T7 replisome include gp2.5 (5 μM dimer), gp4 (100 nM hexamer) and either gp5-Trx (200 U mL^−1^, 80 nM) or Sequenase 2.0 (200 U mL^−1^). The nicking endonucleases (200 U mL^−1^) used in this study are Nb.BssI, Nb.BbvCI and Nt.BbvCI [33]. Reactions (50 μL) were initiated with 10 mM magnesium acetate, incubated at 37 °C for 3 hours, and quenched with a mixture of 20 mM EDTA and proteinase K. Portions of the reactions were visualized by non-denaturing or alkaline agarose gel electrophoresis, and the remainder were prepared for nanopore sequencing.

### Nanopore sequencing

Quenched amplification reactions were purified using Ampure XP magnetic beads with two washes of 70% ethanol (Beckman Coulter, Brea, CA) and eluted in nuclease-free H_2_O (nfH_2_O, Thermo Fisher). DNA concentrations were quantified using a Qubit fluorimeter and the 1x dsDNA HS kit (Thermo Fisher). Each reaction was end-repaired using the Ultra II End Repair/dA-Tailing module (NEB) prior to barcode ligation. A 50 μL solution of 1 μg DNA was combined with 7 μL of the buffer mix and 3 μL of the enzyme mix, incubated at 20 °C for 5 minutes and then 65 °C for 5 minutes. Barcode oligos from the Native Barcoding Kit (2.5 μL, Oxford Nanopore Technologies, ONT) were added to the repaired libraries along with components of the Ultra II Ligation module (1.5 μL ligation enhancer, 34.5 μL master mix, 1.5 μL nfH_2_O, NEB) and ligation occurred in 10 minutes at room temperature. Barcoded libraries were purified using magnetic beads, quantified and pooled in approximately equal proportion by mass.

Adapter ligation for ONT sequencing was accomplished using the Ligation Sequencing Kit. Pooled barcoded libraries (1 μg) were combined to 100 μL with 5 μL barcode adapter mix, 20 μL Quick Ligation reaction buffer (NEB), and 10 uL Quick T4 DNA ligase in nfH_2_O before incubating on the bench for 10 minutes. The final library was purified using magnetic beads and two washes with LFB buffer before elution into EB buffer (ONT) and quantification by Qubit. ONT sequencing was performed using a GridION sequencer, a flow cell and priming kit according to the recommendations of the manufacturer. Basecalling and demultiplexing was performed using the default settings of the MinKNOW software suite.

### Nanopore data analysis

Basecalled and demultiplexed reads were aligned to the λ phage genomic DNA sequence with minimap2 using the default settings for ONT data and excluding secondary alignments (*minimap2 -ax map-ont—secondary = no*) [34]. Number of unaligned reads were counted using samtools (*samtools view -c -F 4*) [35]. The alignment files were sorted (*samtools sort*), converted to a bam file (*-O bam*) and indexed (*samtools index*). Full coverage maps were determined using the complete set of alignments, including multiple alignments from the same read, with bedtools (*bedtools genomecov -d -ibam*) [36]. Coverage maps corresponding to the 5’ or 3’ ends of all alignments were similarly determined using bedtools (*genomecov -d -5* or *-3*). For comparison of read lengths (*sequence_length_template*) and average basecalling quality scores (*mean_qscore_template*), these values were extracted from the sequencing summary text file produced by MinKNOW. Filtering the fastq files by read length or average quality score was accomplished using NanoFilt [37].

Chimeric reads were identified by the supplementary alignment flag produced by minimap2. Chimeric read IDs were listed using pysam and Picard (*FilterSamReads*) was used to extract alignments arising from chimeric reads. Alignments from chimeric reads were converted to the bed file format using bedtools and counted. For reads producing exactly two alignments, the overlap between the alignments was defined in the following way:

$$\%\;overlap=\frac{{End}_{Alignment\;1}-{Start}_{Alignment\;2}}{{Length}_{Shorter\;Alignment}}\;\times \;100$$

where Alignment 1 has its start position toward the left end of the λ phage genomic DNA sequence relative to Alignment 2. If both alignments start at the same position, Alignment 1 was defined as the longer alignment. If the overlap is negative the % overlap is taken to be 0, and if the overlap is greater than 1 the % overlap is taken to be 100.

Plots were generated using the ggplot2 suite in R. For coverage plots, the determined coverage at a given genomic position was divided by the total number of alignments. For kernel density estimate (KDE) plots, the contour levels were linearly spaced, and the scales were excluded. These plots are intended for qualitative identification of clusters of similar reads.

### Capillary electrophoresis assay of gp5 3’ digestion and extension

A fluorescently labeled oligonucleotide corresponding to a region of λ DNA (S4A Fig in S1 File, Integrated DNA Technologies, Coralville, IA) was reacted with either WT gp5 or Δ28 gp5 in the presence or absence of dNTPs. Reactions contained 100 nM oligonucleotide and 0 or 1 mM dNTPs in 1x CutSmart buffer (50 mM potassium acetate, 20 mM Tris acetate, 100 μg/mL bovine serum albumin, 10 mM magnesium acetate, pH 7.9) in a volume of 50 μL. Prior to addition of polymerase, 5 μL of the reaction was removed and combined with 5 μL of quench buffer (20 mM Tris-HCl pH 7.5, 100 mM EDTA, 1% v/v Triton X-100). Reactions were initiated by the addition of polymerase to 20 U mL^−1^, incubated at 37 °C and aliquots were removed and quenched at indicated time points. Quenched samples were diluted 10-fold in nfH2O and labeled products resolved using an Applied Biosystems 3730xl instrument [38].

### Alkaline gel electrophoresis

Samples were prepared for alkaline gel electrophoresis in 6x Purple Loading Dye (NEB). Agarose gels (0.4%) were prepared in alkaline buffer (50 mM NaOH, 10 mM EDTA) and electrophoresis was performed in the same buffer at 1 V cm^-1^ and 4 °C for 16 hours with buffer circulation. Gels were neutralized in 50 mM Tris pH 7.5 buffer, stained with SYBR Gold (Thermo Fisher) and visualized on a Typhoon gel scanner (GE Healthcare).

## Results

**Fig 1:**
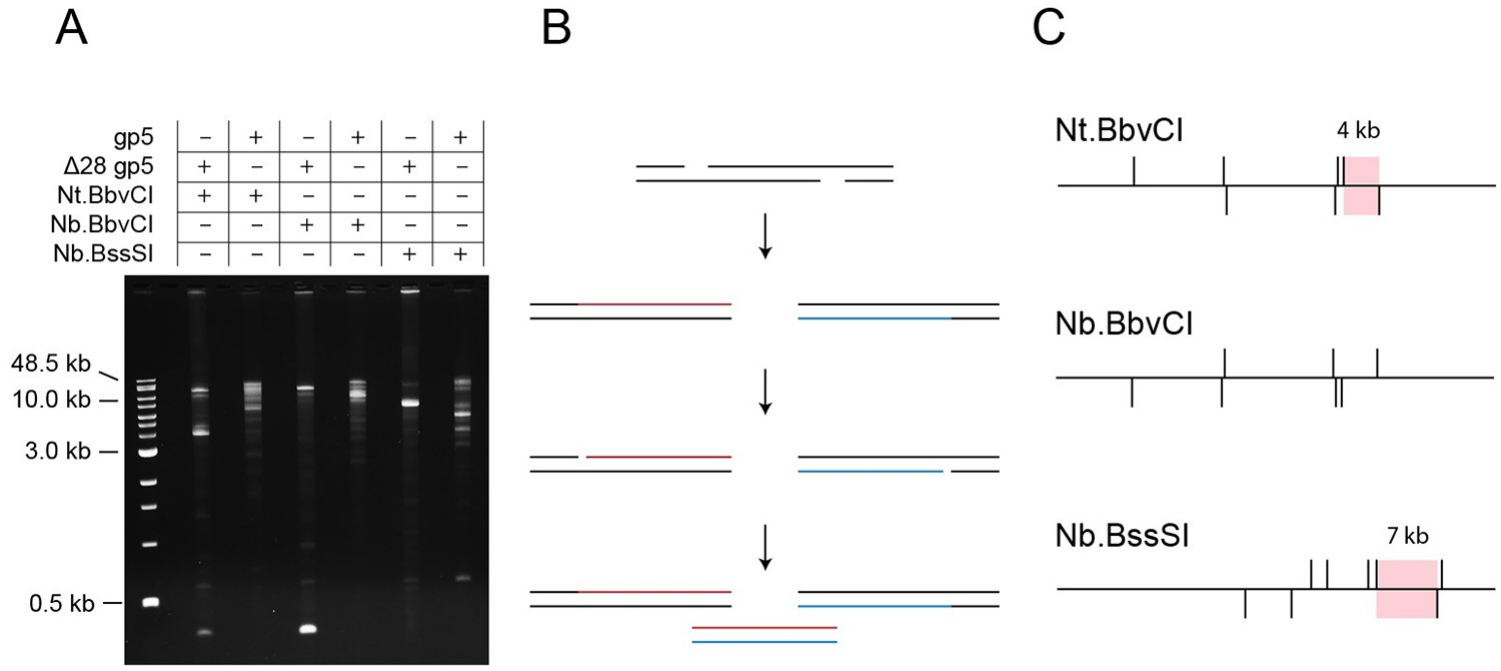
Agarose gel electrophoresis of T7 SDA reactions. (A) Amplicons produced in the reaction of λ DNA with a NEase and either the WT or Δ28 gp5 T7 replisome. (B) A scheme for DNA amplification of a region between two NEase recognition sites. (C) Illustration of NEase recognition sites within the linear λ phage genome. Vertical lines above the horizontal correspond to nicks on the top strand of DNA, and those below to nicks on the bottom strand of DNA. Shaded regions correspond to amplicons.

We first set out to demonstrate strand displacement amplification using the T7 replisome with sequence-specific nicking endonucleases (NEases). Genomic λ phage DNA was selected as a substrate because its size (48.5 kbp) enables robust sequencing depth of reaction products while presenting numerous sites for amplification initiation by available NEases. NEases selectively cut either the top or bottom strand of a double-stranded DNA molecule. The endonucleases Nt.BbvCI and Nb.BbvCI create either a top or bottom strand nick, respectively, at the recognition sequence 5’-CCTCAGC, which occurs seven times in the λ phage genome [39]. The nicking enzyme Nb.BssSI creates a bottom strand nick at three different 5’-CACGAG sites and a top strand nick at five 5’-CTCGTG sites. Each nicking enzyme is expected to produce varied patterns of initiation sites for the T7 replisome within the λ phage DNA substrate. Accordingly, distinct bands of dsDNA amplicons are generated by cycles of nicking and DNA polymerization by the T7 replisome (Fig 1A).

In reactions using the exonuclease deficient T7 replisome, amplicons may be tentatively assigned according to a simple amplification scheme (Fig 1B) and the position of nicking recognition sequences in λ phage DNA. For instance, the principal amplicon in the reaction with Nb.BssSI is about 7 kbp in length. This likely corresponds to a region between top-strand nicking site ^35219^CTCGTG and bottom-strand site ^42416^CACGAG. Similarly, the ~4 kbp band in the reaction with Nt.BbvCI likely corresponds to amplification of the region between top-strand ^31836^CCTCAGC and bottom-strand ^35813^GCTGAGG sites. In all reactions, but particularly those using Δ28 gp5, branched DNA corresponding to an amplification intermediate is trapped in the wells of the agarose gel. Curiously, reactions using the WT T7 replisome produce different amplicons compared to those using the exonuclease deficient polymerase.

### Nanopore sequencing of T7 products

Many of the amplicons observed in Fig 1 are not readily identified by comparison to anticipated template DNA nicking sites. Further, the different product profiles associated with polymerase exonuclease activity do not have an obvious mechanistic explanation. To assign the discrete products apparent in the agarose gel of the SDA reactions, and to elucidate the role of gp5 exonuclease activity in the reaction, we employed nanopore sequencing. Nanopore sequencing yielded sequence reads for each of the six T7 reactions shown in Fig 1. Histograms of read lengths (S1 Fig in S1 File) show clustering for each of the reactions. When Δ28 gp5 polymerase is used, clusters of sequencing read lengths have a straightforward connection to visible bands on the agarose gel. For instance, the 7 kbp amplicon produced with Nb.BssSI is apparent in the nanopore data as a clustering of sequencing read lengths of 7 kilonucleotides (knt). Nearly all of the sequencing reads could be aligned to λ phage DNA using the minimap2 algorithm (Table 1).

**Table 1:** Nanopore sequencing statistics for T7 SDA reactions.

	Nt.BbvCI	Nt.BbvCI	Nb.BbvCI	Nb.BbvCI	Nb.BssSI	Nb.BssSI
Polymerase	Δ26 gp5	gp5	Δ26 gp5	gp5	Δ26 gp5	gp5
Reads	317083	753050	457596	953507	210457	192085
Read Length N50	5377	6986	7878	9303	7106	7098
Q>10 Reads^b^	234538	498176	294340	651211	137052	101687
Unaligned Reads	2013	26846	1037	2266	1210	563
Alignments	351132	877706	523911	252373	258166	256629
Sup. Alignments^c^	36062	151502	67352	155232	48919	65107
Chimeric Reads^d^	32627	128674	60588	128859	43977	54683

^*b*^Reads with average basecalling quality score over 10.

^*c*^Number of supplementary alignments found by minimap2

^*d*^Determined by counting the number of reads with at least one supplementary alignment.

**Fig 2:**
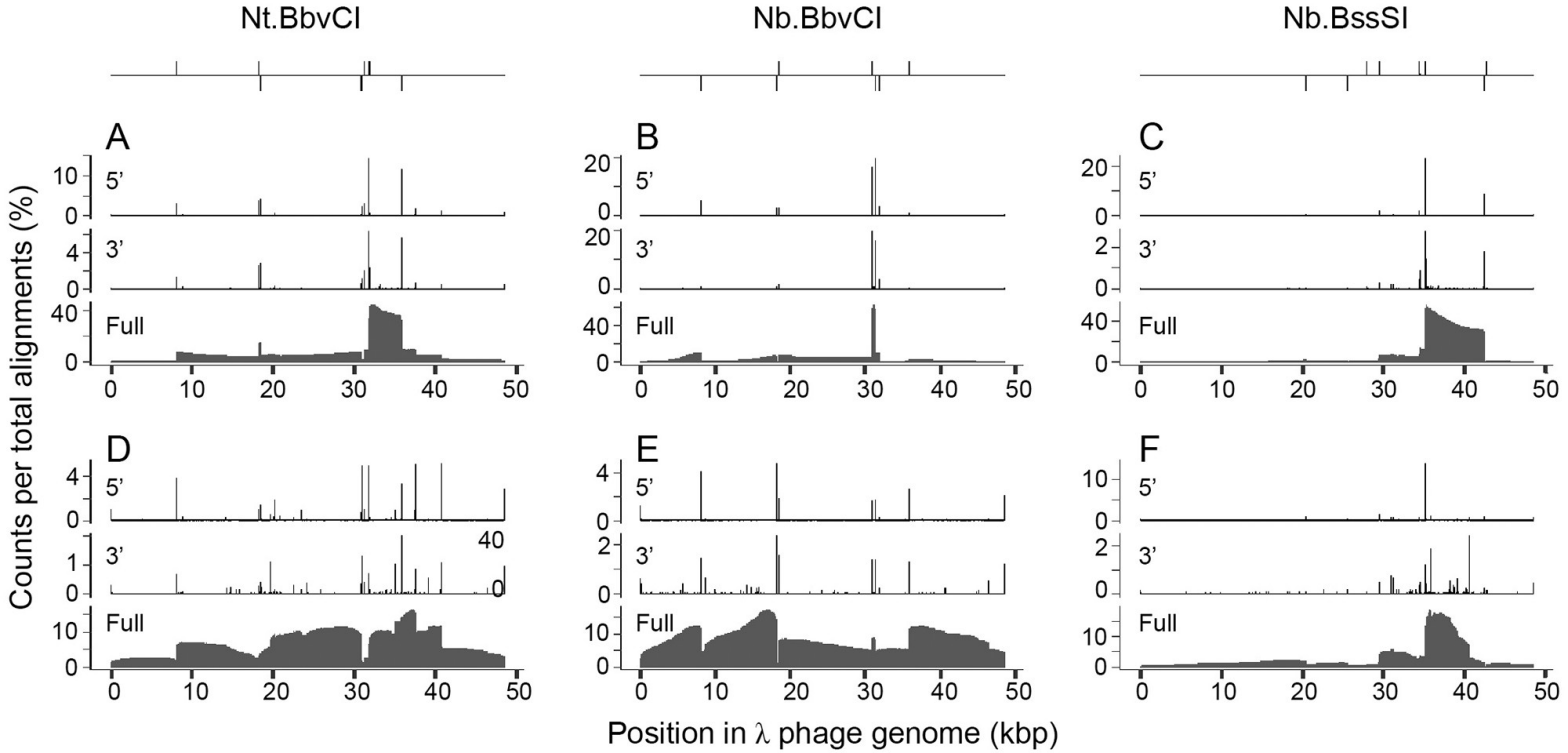
Coverage maps from nanopore sequencing data for T7 SDA reactions. Each panel shows 3’, 5’ and full coverage for all alignments produced by minimap2 including those from chimeric reads. Counts per total alignments reflect the coverage at each nucleotide divided by the number of alignments. At top are diagrams showing nicking recognition sites within the λ phage genome, where lines above the horizontal correspond to anticipated top strand nicks, and those below to bottom strand nicks. The nicking enzyme and polymerase used in each panel are (A) Nt.BbvCI and Δ28 gp5; (B) Nb.BbvCI and Δ28 gp5; (C) Nb.BssSI and Δ28 gp5; (D) Nt.BbvCI and WT gp5; (E) Nb.BbvCI and WT gp5; and (F) Nb.BssSI and WT gp5.

Coverage maps of the 3’ and 5’ ends of the alignments, as well as full alignment intervals, are shown in Fig 2 and form the basis for a mechanistic understanding of the T7 SDA reaction. At top are diagrams showing the position and orientation of NEase recognition sites, which broadly correspond to the 5’ and 3’ termini of alignments. 5’ ends are generated in the SDA reaction by the nicking enzyme and have lower heterogeneity compared to 3’ ends which result from polymerase activity. For Nt.BbvCI, the 5’ coverage map reveals several off-target sites that differ from the cognate recognition sequence by a single nucleotide: ^20145^GCTCAGG, ^40798^GCTTAGG and ^37586^GCTTAGG. The 5’ coverage maps demonstrate that amplified dsDNA is generated by repeated cycles of nicking and strand-displacing DNA polymerization. Reactions with the exonuclease deficient T7 replisome (Fig 2A–2C) show prominent regions of high coverage between sequential top and bottom-strand nicking recognition sites as expected from the amplification scheme of Fig 1B.

**Fig 3:**
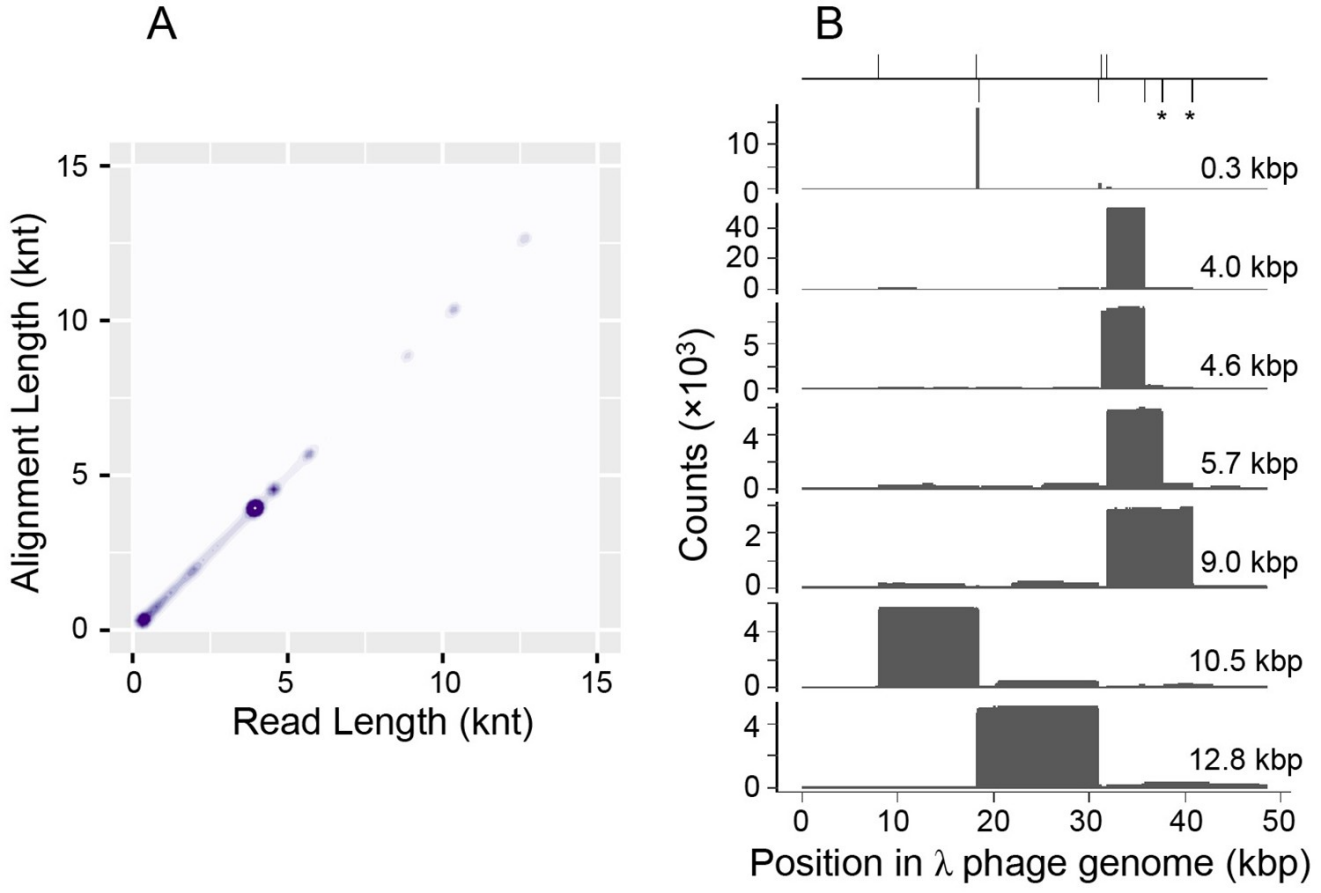
Identification of dsDNA amplicons in a T7 SDA reaction. Data are shown for the reaction of Δ28 gp5 and Nt.BbvCI. (A) Kernel density estimate plot of read length against alignment length. Reads were filtered to have average basecall quality greater than 10. Only one alignment is considered per read. (B) Coverage maps of reads with specified lengths. At top is a diagram showing recognition sites for Nt.BbvCI in the λ phage genome. Two additional sites of sequence GCTTAGG are denoted with asterisks.

Identification of dsDNA amplicons in the reaction of Δ28 gp5 T7 replisome and Nt.BbvCI is demonstrated in Fig 3. A density plot of alignment length against read length (Fig 3A) shows isolated clusters along the diagonal. These represent groups of reads of similar size which make a single uninterrupted alignment to λ phage DNA. Clusters were selected by read length and used to produce the coverage maps in Fig 3B. The dominant 4 kbp amplicon aligns as anticipated between sequential top and bottom strand Nt.BbvCI recognition sites. Longer amplicons up to 13 kbp correspond to amplification between initiation sites with one intervening nicking recognition site. The longest amplification product is a single 23 knt read that aligns as a reverse complement between ^8014^T and ^30920^A, covering two Nt.BbvCI recognition sequences. A similar analysis for amplicons produced with Nb.BbvCI and Nb.BssSI is shown in S2 Fig in S1 File. These amplicons represent the prominent bands on the agarose gel (Fig 1) produced by the exonuclease deficient T7 replisome.

### Template switching in T7 reactions

The distinct product profile of the WT T7 replisome suggests that the exonuclease activity of WT gp5 promotes a different mechanism for dsDNA amplification. For many of the sequencing reads in all of the T7 reactions, supplementary alignments are found by minimap2 (Table 1). These represent reads that make at least two distinct alignments to different regions of the template DNA, called chimeric reads and commonly observed in multiple-displacement amplification reactions. Minimap2 does not always produce multiple alignments for a chimeric read and computational tools available for handling chimeras generated in multiple-displacement amplification reactions may not be appropriate [40, 41]. For all of the T7 reactions, analysis of the identified chimeric reads indicates that inverted repeats (IRs) are dominant (S3 Fig in S1 File). The overwhelming majority of chimeric reads produce exactly two alignments. Of the reads that make two alignments, the two alignments are almost always on opposite strands, and with extensive overlap.

**Fig 4:**
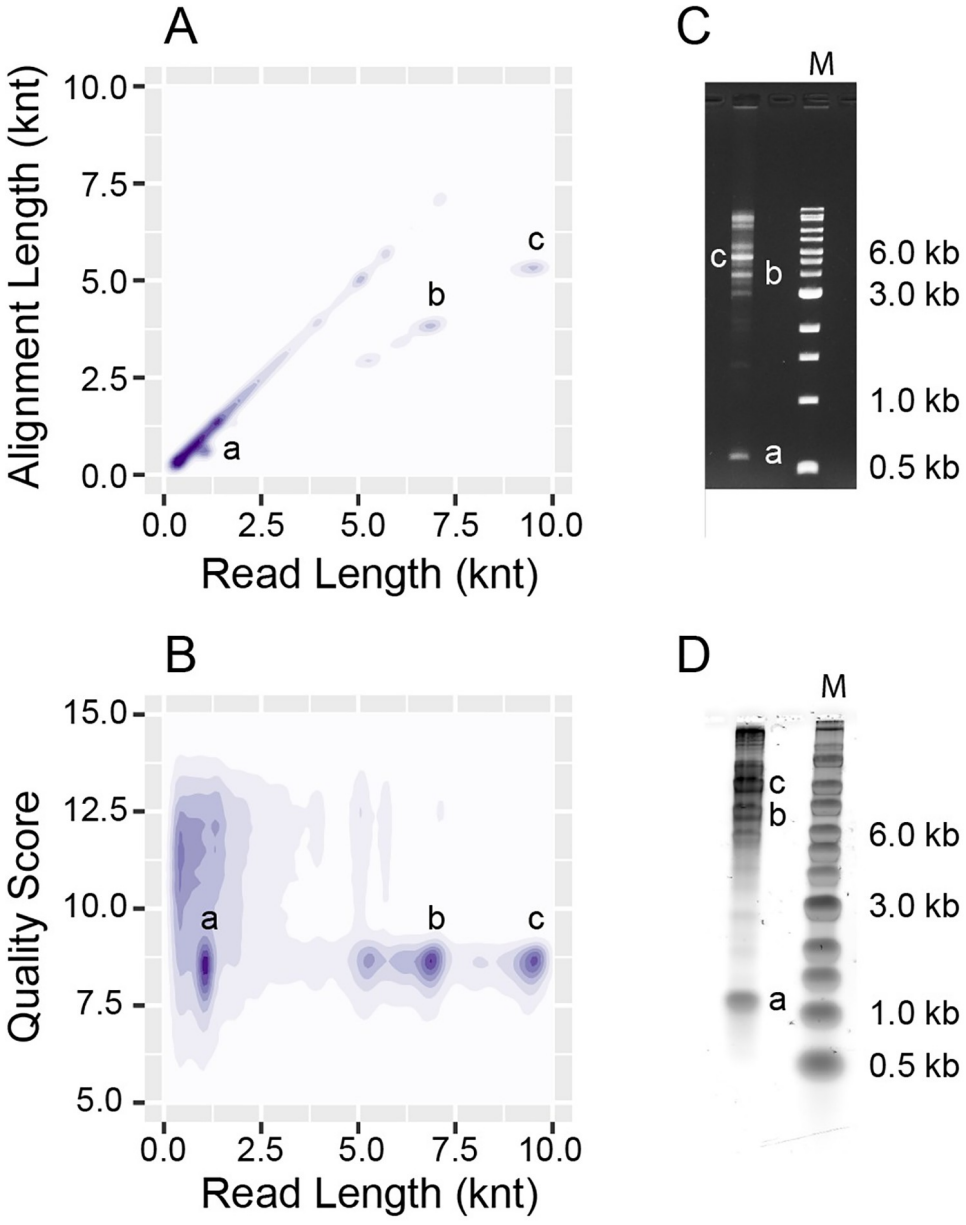
Amplification of hairpins in a T7 SDA reaction. Data are shown for the reaction of WT gp5 and Nt.BssSI. (**A**) Kernel density estimate (KDE) plot of read length against alignment length. Only one alignment is considered per read. (**B**) KDE plot of read length against average read quality score. (**C**) Non-denaturing agarose gel of the reaction products stained with ethidium bromide. (**D**) Denaturing alkaline agarose gel of the reaction products. In each panel, clusters of amplified hairpin DNA molecules are labeled with lower case letters.

The reaction with Nb.BssSI best demonstrates the presence of IR chimeric reads in the sequencing data. Clusters of chimeric reads are apparent as off-diagonal features in a density plot of alignment length against read length (Fig 4A). For all these clusters the alignment length is about half of the read length, suggestive of a complete inverted repeat sequence. Inverted repeats present a challenge for nanopore sequencing, and a clear association is found between read length and read quality within this data set (Fig 4B). When an IR sequence is passed through the nanopore, the second instance of the repeat gives systematically lower quality data than the first likely due to refolding as the strand translocates through the pore [42]. Low basecalling quality impedes alignment of the data and presents a challenge to quantitative analysis of this type of chimeric read using nanopore sequencing. For this reason, the 5’ and 3’ coverage maps of all alignments (Fig 2) represent only one alignment within a chimeric read. The sequencing data suggest that a large fraction of the amplicons produced by the WT T7 replisome are inverted repeats. Denaturing alkaline gel electrophoresis was used to investigate the structure of amplicons in the reaction with Nb.BssSI (Fig 4C and 4D). Mobility of the unfolded strands relative to a marker of linear dsDNA under denaturing or non-denaturing conditions demonstrate that the IRs apparent in the sequencing data correspond to DNA hairpins.

Strand-displacement amplification using the T7 replisome primarily produces two types of dsDNA products: linear molecules with two blunt ends and hairpins. Exonuclease deficient T7 replisome tends to make linear dsDNA whereas the WT T7 replisome produces a greater proportion of hairpins, though both complexes make both products to some degree. Linear amplicons correspond to regions of the template between appropriately oriented nicking sites and may be relatively long, up to 23 kbp and regularly over 10 kbp.

## Discussion

### Polymerase 3’ exonuclease activity and template switching in T7 SDA

**Fig 5:**
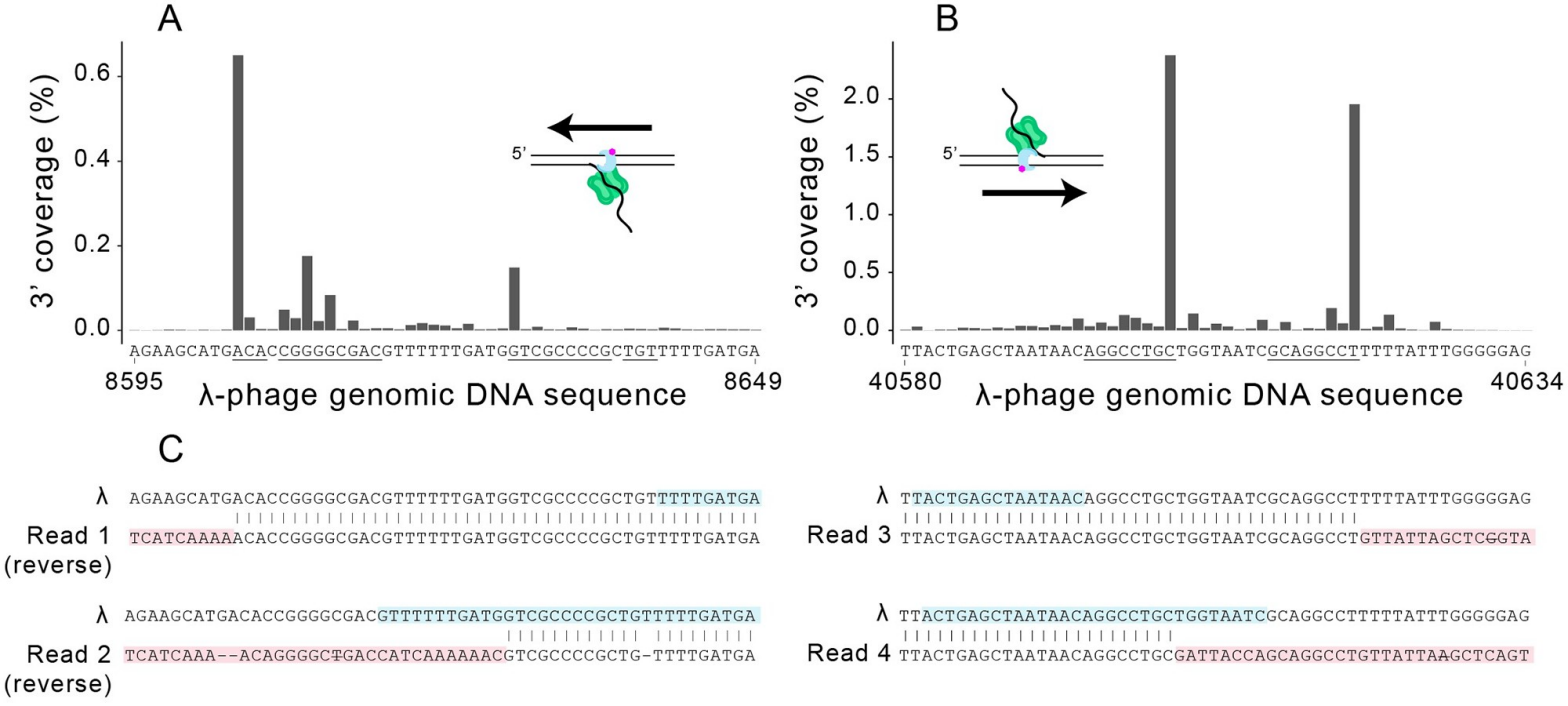
Inverted repeats are sites for template switching. (A) 3’ Coverage over the region 8695–8649 in the reaction with Nb.BbvCI. Strand displacing polymerization may only be initiated from the right. (B) 3’ Coverage over the region 40580–40634 in the reaction with Nb.BssSI. There are nicking recognition sequences on both sides of this region, but polymerization is principally initiated from the left. (C) Selected reads showing template switching. Representations of the full alignments for selected reads are shown in S5 Fig in S1 File.

Analysis of T7 reactions using either WT gp5 or Δ28 gp5 in the context of the replisome demonstrates that 3’ exonuclease activity is associated with amplification of DNA hairpins. An explanation for this observation is found in the 3’ coverage of alignments in the WT T7 replisome reactions (Fig 2). In the Δ28 gp5 reactions, prominent positions of 3’ coverage correspond to nicking recognition sites, as expected for linear dsDNA molecules amplified from the region between two nicks. In the WT gp5 reactions, additional sites of high 3’ coverage, representing the end of an alignment, are apparent. In fact, these features serve as markers for short inverted repeated (IR) sequences within the λ phage DNA template (Fig 5A and 5B). Inverted repeat sequences may anneal and provide an extendable substrate for the polymerase to produce a chimeric DNA molecule. The features at IR sequences are best explained by a mechanism in which WT gp5 digests the 3’ end of a ssDNA intermediate until it reaches a duplex IR region, and then extends. This model was tested using a ssDNA substrate that forms a partial hairpin with a 6 nt non-complementary tail. This substrate is inert to Δ28 gp5 but may be converted to the full hairpin by WT gp5 (S4 Fig in S1 File). A recent analysis of PCR errors by PacBio high accuracy sequencing demonstrated that polymerase 3’ exonuclease activity was associated with increased template switching at IRs [43]. IR-mediated template switching by the WT T7 replisome represents an extreme example of this phenomenon owing to the high exonuclease activity of this polymerase and stable annealing of the inverted repeat at 37 °C.

Chimeric hairpin reads in the T7 reactions may arise from two different mechanisms: production of ssDNA which folds on itself for subsequent extension, or polymerase dissociation followed by branch migration. Both mechanisms likely contribute to hairpin production, but the sequencing data indicate that template switching of the nascent chain to the displaced strand is dominant. The full coverage profiles (Fig 2), higher near the site of polymerase initiation, show asymmetry that indicates template switching occurs before ssDNA is expected to be displaced by run-off polymerization. Production of short ssDNA could result from replisome stalling followed by another round of nicking and extension from the initiation site, but this explanation is unlikely considering that nicking enzyme activity is typically limiting in SDA reactions, particularly for Δ28 gp5 [20, 23].

Stronger evidence for intermolecular template switching is apparent by inspecting individual chimeric reads. Dot plot representations of BLAST alignments for four chimeric IR reads are shown in S5 Fig in S1 File. The sites of template switching for each of the four reads are shown in Fig 5C. Two of the reads have alignments which end at the second instance of the repeat and could arise from intramolecular priming. The other two reads switch templates at the first instance of the repeat. This is inconsistent with hairpin production via ssDNA and demonstrates a cruciform-structure intermediate encountered during amplification.

**Fig 6:**
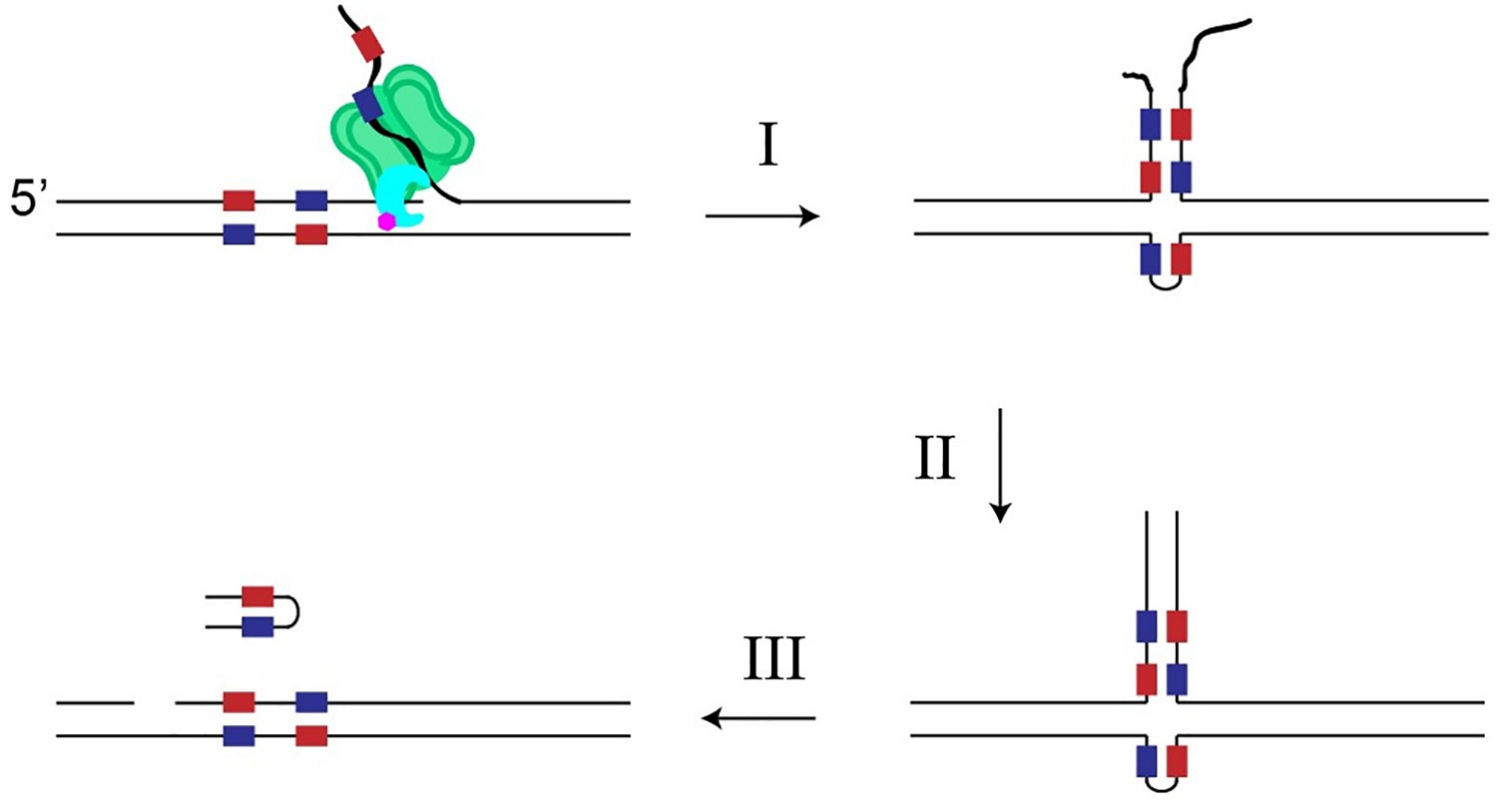
A scheme for amplification of hairpin DNA. (I) Replisome dissociation and DNA branch migration produce a cruciform structure at a suitable inverted repeat sequence, depicted as red and blue blocks. (II) 3’ exonuclease activity of WT gp5 enables extension of the cruciform intermediate. Extension may restart at either the first or second instance of the inverted sequence. (III) Another round of nicking allows for hairpin release. Hairpin release may be assisted by another round of polymerization from the DNA nick.

A mechanism for template switching in T7 reactions is given in Fig 6. The first steps involve dissociation of the T7 replisome to produce a branched DNA molecule. Branch migration can occur, producing a free 3’ end from the recently synthesized chain. If replisome dissociation occurred near an inverted repeat, isomerization of the DNA can produce a ‘cruciform’ structure. For Δ28 gp5, with no 3’ exonuclease activity, these intermediates are a dead end. Gradual isomerization back to the branched structure and reloading of the polymerase can then continue the on-target amplification. If the DNA polymerase has 3’ exonuclease activity, the free 3’ may be digested back to the double-stranded region, at which point template switching will occur. Subsequent nicking at the initiation site and branch migration will then produce the free dsDNA hairpin.

An analogous mechanism for template switching has been proposed for reactions using an oxidized form of T7 gp5 which like Δ28 gp5 has inherent strand-displacement activity [44]. Acting alone, this polymerase extends only about one hundred nucleotides before switching to the displaced strand. Previous work has shown that gp4 is sufficient to allow WT gp5 to initiate from nicked DNA [18], that gp4 is necessary for amplification of long DNA concatemers by Δ28 gp5 [14], and that gp4 enhances the processivity of gp5 [45]. We expect that gp4 reduces the degree of template switching in T7 SDA and extends the size of attainable products. Protein-protein interactions among T7 gp2.5, gp4, gp5 and *E*. *coli* thioredoxin are essential for the function of the T7 replisome and likely confer advantages to its use in isothermal amplification methods.

Chimeric reads in next generation sequencing data are also observed in whole genome amplification methods, namely multiple displacement amplification (MDA) reactions using ϕ29 DNA polymerase [27, 41]. In contrast to the T7 reactions, MDA relies on hexameric oligonucleotides of random sequence to initiate amplification [46]. Highly branched structures, with multiple strands of ssDNA in close proximity, are possible and enable template switching mechanisms which produce chimeric reads other than inverted repeats. Recent analysis of MDA using long-read sequencing methods demonstrated a high proportion (up to 50% of reads in some reactions) of inverted repeats, and a computational method was presented to identify and collapse long-read IRs [40]. As in MDA, inverted repeat chimeric reads are produced in the T7 reactions (S3 Fig in S1 File). In general, whole-genome amplification methods for long-read sequencing which produce long ssDNA intermediates may be expected to produce chimeric reads. Computational tools which use self-alignment of long reads to identify chimerism, while distinguishing natural duplicate sequences, are appropriate to avoid systematic errors in genome assembly from long reads.

### Applications and extensions of T7 SDA

Several shortcomings of T7 replisome strand-displacement amplification limit the utility of the method in its present form. In general, linear amplification methods, in which the product DNA cannot act as a substrate, have slower kinetics and limited sensitivity compared to exponential methods like PCR or MDA [7]. Linear amplification has been using in a single-cell sequencing technique to avoid bias introduced by PCR and increase sequencing accuracy [47]; however, Δ28 gp5 has a reduced fidelity relative to WT gp5 owing to the mutations in the proof-reading exonuclease domain [48]. A possibility is that the WT T7 replisome could be used with a non-specific nicking endonuclease, e.g. CviPII [49], for whole-genome amplification in analogy to MDA. Additional experiments are needed to evaluate the utility of T7 SDA in unbiased, non-specific amplification for long- and short-read sequencing methods.

An extension of this technique is the use of customizable nicking endonucleases, such as *Streptococcus pyogenes* D10A Cas9 or *Claustridium butyricum* Argonaute [50–52]. Single-turnover activity of Cas9 has been used to initiate exponential strand-displacement amplification reactions [53]. Unfortunately, both enzymes are known to have an extremely slow rate of dissociation from DNA following cleavage. Some Cas9 homologues show faster rates of enzymatic turnover than the *S*. *pyogenes* enzyme and other enzymes such as RNA polymerase may displace Cas9 to promote turnover [54, 55]. Discovery of a customizable nicking enzyme with fast turnover would greatly enhance the applications of T7 SDA. As longer DNA molecules find use in synthetic biology and sequencing applications, there is a need for novel and creative amplification strategies which will likely involve enzymes beyond DNA polymerases.

## Supporting information

S1 File (DOCX)
