# Supplementary material for: Chimeric DNA byproducts in strand displacement amplification using the T7 replisome
Source: PLoS One. 2022 Sep 19;17(9):e0273979. doi: 10.1371/journal.pone.0273979 (PMC9484634; doi:10.1371/journal.pone.0273979)
Supplement: S1 File — (DOCX) [file pone.0273979.s001.docx]

**Chimeric DNA Byproducts in Strand Displacement Amplification Using the T7 Replisome**

Dillon B. Nye^1.^and Nathan A. Tanner^1^

^1^ Nucleic Acid Replication Division, New England Biolabs Inc., 240 County Road, Ipswich, MA

**Supporting Information**

Fig S1: Nanopore sequencing read length histograms for the T7 SDA reactions.

Fig S2: Identification of dsDNA amplicons in Δ28 gp5 SDA reactions.

Fig S3: Inverted repeats in T7 SDA reactions.

Fig S4: Capillary electrophoresis data showing extension of a partial hairpin by WT gp5.

Fig S5: Dot plot representations of BLAST alignments for selected reads.

Supplementary Methods: Protein purification information

| 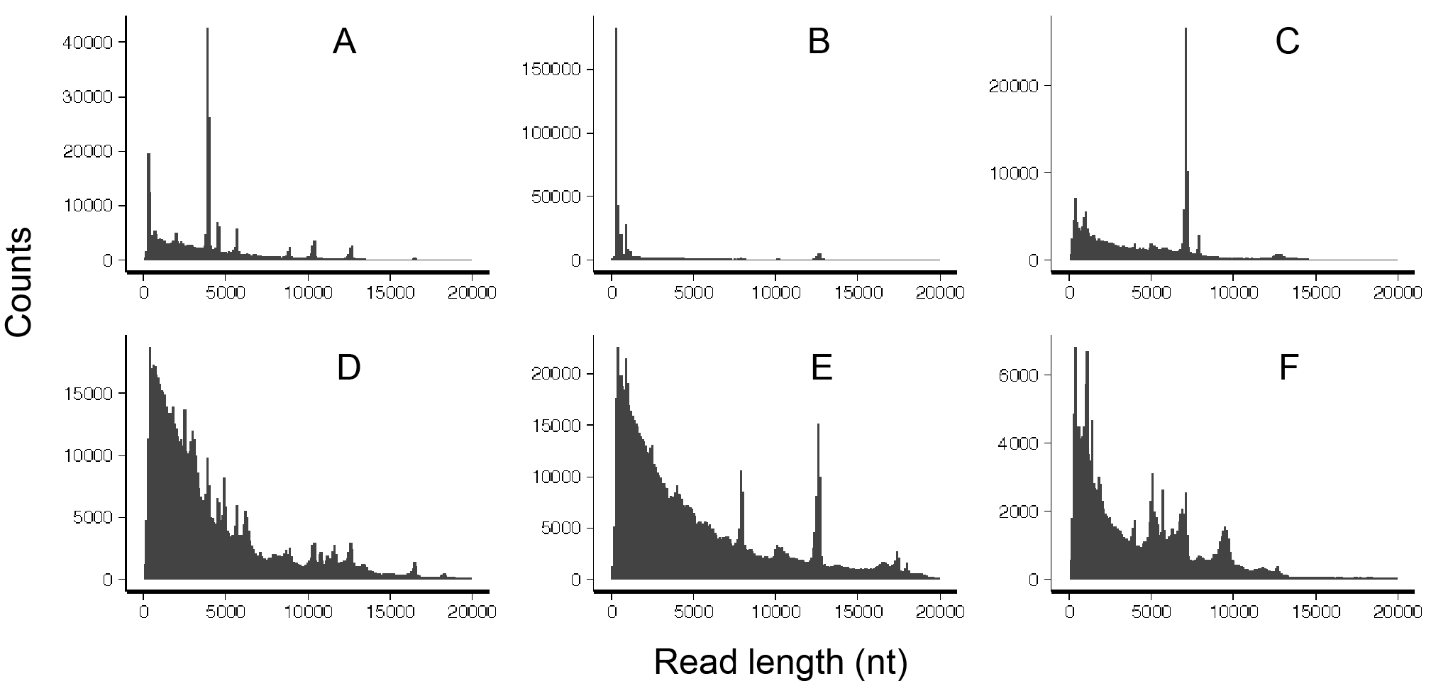 |
| --- |
| **Fig S1**. **Nanopore sequencing read lengths for T7 SDA reactions**.  Histograms showing read lengths for the T7 SDA reactions. Bin width is 100 nt. For reactions using Δ28 gp5 but not WT gp5, the read lengths have an obvious connection to bands observed by gel electrophoresis. (**A**) Nt. BbvCI and Δ28 gp5; (**B**) Nb. BbvCI and Δ28 gp5; (**C**) Nb. BssSI and Δ28 gp5; (**D**) Nt. BbvCI and WT gp5; (**E**) Nb. BbvCI and WT gp5; and (**F**) Nb. BssSI and WT gp5. |

| 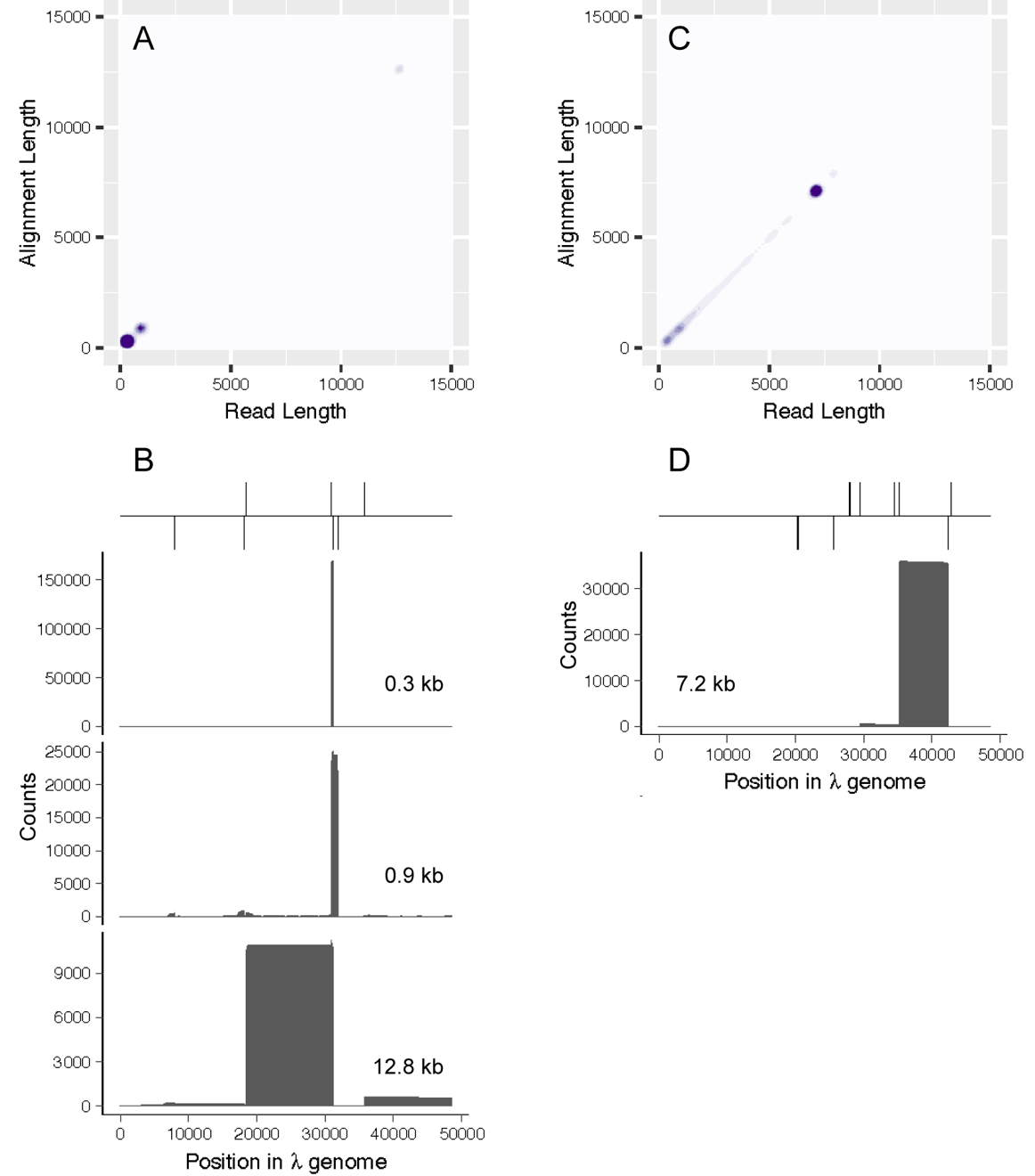 |
| --- |
| **Fig S2.** **Identification of dsDNA amplicons in Δ28 gp5 SDA reactions**.  Kernel density estimate plots for amplicons produced in the reactions of (A) Δ28 gp5 and Nb. BbvCI and (C) Δ28 gp5 and Nb. BssSI. Reads were filtered to have average basecalling quality score greater than 10 and only one alignment is considered per read. Reads were filtered by length and used to generate the full coverage histograms displaying linear dsDNA amplicons in the reactions of (B) Δ28 gp5 and Nb. BbvCI and (D) Δ28 gp5 and Nb. BssSI. |

| 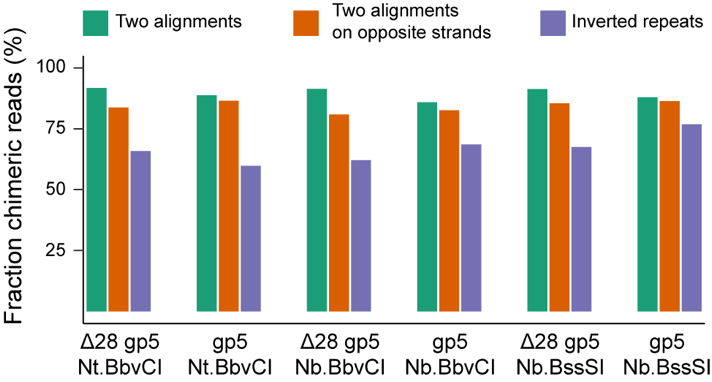 |
| --- |
| **Fig S3. Inverted repeats in T7 SDA reactions**.  Proportions of chimeric reads that make exactly two alignments to the λ phage DNA template (green), on opposite strands (orange) with at least 80% overlap (purple) are shown. The corresponding T7 SDA reactions are labeled at the bottom. The majority of chimeric reads in all reactions correspond to inverted repeats. |

| 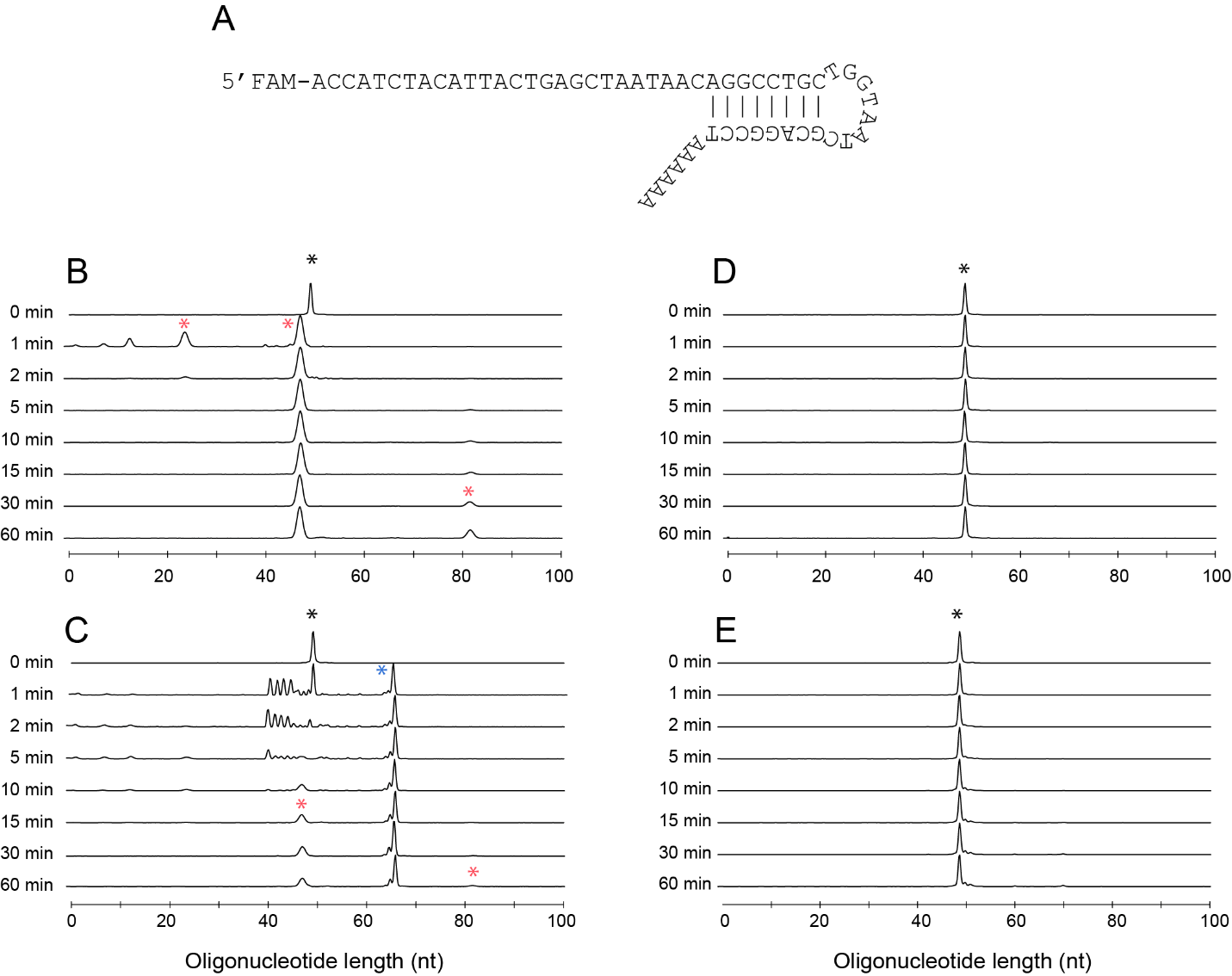 |
| --- |
| **Fig S4. Capillary electrophoresis data showing extension of a partial hairpin by WT gp5.**  (A) Depiction of a folded form of the fluorescently labeled substrate used in this experiment. Shown are time-course traces in reactions of the substrate with (B) WT gp5 and no dNTPs; (C) WT gp5 and 1 mM dNTPs; (D) Δ28 gp5 and no dNTPs; and (E) Δ28 gp5 and 1 mM dNTPs. Black asterisks denote the unreacted oligonucleotide. Red asterisks denote digested FAM labeled oligonucleotides. Blue asterisks denote the extended hairpin products. Elution times are imperfectly converted to nucleotide lengths by comparison to internal standards. Values for intensities are not shown as only the elution time is relevant. |

| 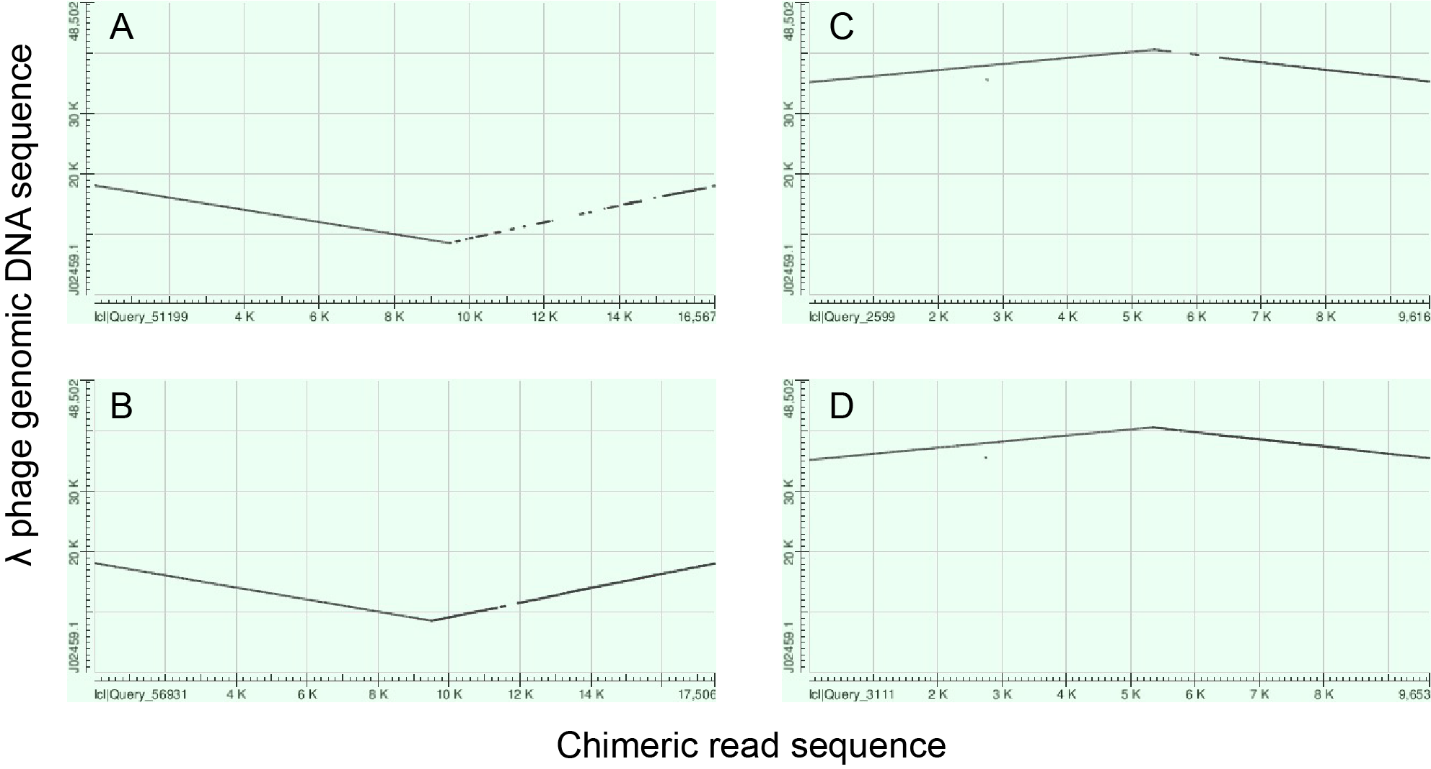 |
| --- |
| **Fig S5.** **Dot plot representations of BLAST alignments for selected chimeric reads.**  The BLAST-N algorithm was used and figures were taken directly from the BLAST webserver. The reads shown here correspond to those in Figure 5. (**A**) Read 1 (reverse) and (**B**) Read 2 (reverse) come from the reaction of WT gp5 and Nb. BbvCI, and (**C**) Read 3 and (**D**) Read 4 from the reaction of WT gp5 and Nb. BssSI. Note that the second portion of the read suffers from low basecalling quality which produces a number of deletions and impairs alignment. |

**Supplementary Methods**

**Purification of T7 gp4 and gp2.5.** Cells containing the T7 gp2.5 or T7 gp4 expression vector were grown in LB media to an optical density of 0.7 and induced with 0.5 mM isopropyl β-d-1-thiogalactopyranoside (IPTG, Gold Biotechnology, St. Louis, MO) at 37 °C for 2 hours (gp2.5) or 16 °C overnight (gp4). Following centrifugation (3300 xg, 20 minutes, 4 °C) cell pellets were stored at -20 °C. Frozen cell pellets were then thawed and resuspended in buffer A (50 mM Tris HCl pH 7.5, 1 mM EDTA, 1 mM DTT, 5% glycerol). Cell breakage was performed with a microfluidizer and the lysate clarified by centrifugation (20000 xg, 30 min, 4 °C) prior to filtration and loading on a DEAE column (Sartorious, Göttingen, DE) equilibrated in buffer A. Bound protein was eluted in a 0 – 1.0 M NaCl gradient and fractions containing gp2.5 or gp4 were diluted to 0.1 M NaCl in Buffer A, loaded onto a HiPrep Heparin FF 16/10 column and eluted in a 0.1 – 1.0 M NaCl gradient. Fractions containing gp2.5 were pooled and solid ammonium sulfate (Sigma) was added to 65% saturation. Ammonium sulfate precipitation was excluded in the purification of gp4. Precipitated protein was collected by centrifugation (20000 xg, 20 min, 4 °C), dissolved in buffer A and loaded onto a HiPrep Q FF 16/10 column (GE Healthcare). Ammonium sulfate precipitation was excluded in the purification of gp4, which was diluted in Buffer A prior to loading onto the Q column. Purified protein was eluted in a 0 – 1.0 M NaCl gradient, concentrated using a 10 kDa-cutoff centrifugal column and finally dialyzed overnight into storage buffer (50 mM Tris HCl pH 7.5, 0.1 mM EDTA, 1 mM DTT, 50% glycerol). The final gp2.5 and gp4 products were stored at -20 °C until use and judged to be >95% pure by SDS-PAGE. Protein concentration was determined using a Bradford assay.^29^
